# Characterization of Viral Interference in *Aedes albopictus* C6/36 Cells Persistently Infected with Dengue Virus 2

**DOI:** 10.3390/pathogens12091135

**Published:** 2023-09-06

**Authors:** Aurora Montsserrat González-Flores, Mariana Salas-Benito, Victor Hugo Rosales-García, Paola Berenice Zárate-Segura, Rosa María Del Ángel, Mónica Ascención De Nova-Ocampo, Juan Santiago Salas-Benito

**Affiliations:** 1 Centro de Estudios Científicos y Tecnológicos del Estado de Tlaxcala, Tlaxcala 90491, Tlax., Mexico; aurora.monserrat.gonzalez@cecyte.edu.mx; 2 Escuela Nacional de Medicina y Homeopatía, Instituto Politécnico Nacional, Mexico City 07320, Mexico; msalasb@ipn.mx (M.S.-B.); mdenova@ipn.mx (M.A.D.N.-O.); 3 Laboratorios Centrales, Centro de Investigación y de Estudios Avanzados del IPN, Mexico City 07360, Mexico; vrosales@cinvestav.mx; 4 Escuela Superior de Medicina, Instituto Politécnico Nacional, Mexico City 11340, Mexico; pzarates@ipn.mx; 5 Departamento de Infectómica y Patogénesis Molecular, Centro de Investigación y de Estudios Avanzados del IPN, Mexico City 07360, Mexico; rmangel@cinvestav.mx

**Keywords:** arboviruses, viral interference, viral persistence, PIWI-RNA pathway, mosquitoes

## Abstract

Arboviruses are an important group of pathogens that cause diseases of medical and veterinary concern worldwide. The interactions of these viruses with their host cells are complex, and frequently, the coexistence of two different viruses in the same cell results in the inhibition of replication in one of the viruses, which is a phenomenon called viral interference. This phenomenon can be exploited to develop antiviral strategies. Insect cell lines persistently infected with arboviruses are useful models with which to study viral interference. In this work, a model of C6/36-HT cells (from *Aedes albopictus* mosquitoes) persistently infected with Dengue virus, serotype 2, was used. Viral interference was evaluated via plaque and flow cytometry assays. The presence of heterotypic interference against the other serotypes of the same virus and homologous interference against yellow fever virus was determined; however, this cell line did not display heterologous viral interference against Sindbis virus. The mechanisms responsible for viral interference have not been fully elucidated, but small RNAs could be involved. However, the silencing of Ago3, a key protein in the genome-derived P-element-induced wimpy testis pathway, did not alter the viral interference process, suggesting that viral interference occurs independent of this pathway.

## 1. Introduction

Viruses are strict intracellular infectious agents that require host cell machinery to replicate. When two different viruses infect the same cell, the infection of each virus can follow different courses depending on the virus. Viral accommodation implies that both viruses are equally able to replicate [1]. However, the phenomenon where one virus inhibits the replication of another virus is called viral interference or superinfection exclusion [2,3]. Viral interference could be homologous when the primary and secondary viruses are closely related or heterologous when the viruses belong to different families [3,4]. In viruses that are very closely related, such as dengue virus (DENV) serotypes, this homologous viral interference is referred to as heterotypic viral interference [5]. The mechanism underlying viral interference is not completely understood [2,4].

Arbovirus, a term used to describe viruses that are transmitted by arthropods such as mosquitoes and ticks, includes a vast group of viruses belonging to the families *Togaviridae*, *Flaviviridae*, *Bunyaviridae*, *Rhabdoviridae*, and *Reoviridae*, many of which are responsible for diseases in humans and animals. Some of these viruses are DENV, Murray Valley encephalitis virus (MVE), yellow fever virus (YFV), West Nile virus (WNV), Japanese encephalitis virus (JEV), Saint Louis encephalitis virus (SLE), and Zika virus (ZIKV) in the Flaviviridae family; Semliki forest virus (SFV), chikungunya virus (CHIKV), Sindbis (SINV), and Ross River virus (RRV) in the Togaviridae family; and Bunyamwera, Banzi, and La Crosse virus (LACV) in the Bunyaviridae family [6].

Several arboviruses are able to establish a long-term infection, referred to as a persistent infection, in their vectors. This type of infection has been established in different mosquito cell lines, and these cell lines serve as useful models to study several aspects of persistent infections, including viral interference [3,4,7,8,9,10,11,12,13,14,15,16,17,18,19,20]. In our laboratory, we established a C6/36-HT cell line persistently infected with a DENV-2 serotype, named C6-L [21]. In this work, we characterized the viral interference displayed by this cell line and explored one possible mechanism.

## 2. Materials and Methods

### 2.1. Cells

*Aedes albopictus* C6/36-HT cells [22] adapted to grow at 35 °C [23], C6-L cells persistently infected with DENV 2 [21], and BHK-21 cells were cultured using MEM (Gibco) supplemented with vitamins (Gibco), 10% fetal bovine serum, 0.034% sodium bicarbonate (JT Baker), and antibiotics (100 μg/mL streptomycin and 100 U/mL penicillin) (Sigma, St. Louis, MO, USA). Vero cells were cultured in high glucose D-MEM (Invitrogen, Waltham, MA, USA) supplemented with 10% fetal bovine serum, 0.59% HEPES (Gibco), 0.37% sodium bicarbonate (JT Baker), nonessential amino acids (Gibco), 2 mM sodium pyruvate (Gibco), and antibiotics (100 μg/mL streptomycin and 100 U/mL penicillin) (Sigma).

C6/36 and C6-L cells were grown in a CO_2_-free incubator (Lab Line), and BHK-21 and Vero cells were grown in an incubator (Lab Line) with 5% CO_2_.

### 2.2. Viruses

Reference strains of all DENV serotypes were used in the experiments (DENV-1 Hawaii, DENV-2 New Guinea C, DENV-3 H-87, and DENV-4 H-241). For SINV, the Ar-339 strain (ATCC VR-1248) was used. For YFV, the 17D-204 strain was used. All viruses were stored at −80 °C in an ultra-low freezer (Sanyo Vip Series MDF-U53VC).

### 2.3. Plaque Assay

To titer the DENV and SINV viruses, a plaque assay in BHK-21 cells was performed as previously described [21]. DENV plaque assays were stained with naphthol blue black (0.1% naphthol blue black, 0.165 M sodium acetate, and 6% acetic acid) for 15 min at room temperature at 5 days post infection. The same procedure was carried out for SINV plaque assays but at 3 days post infection.

YFV was titered via plaque assay performed in Vero cells as previously described [24,25].

### 2.4. RT-PCR Assay

To confirm the presence of the DENV-2 genome in the persistently infected C6-L cell line, RT-PCR was performed using the RT-PCR Access kit (Promega, Madison, WI, USA) as previously described [21]. Briefly, total RNA from C6-L cell monolayers was purified using TRIzol^®^ reagent (Invitrogen) according to the manufacturer’s instructions and treated with RNAse-free DNAse RQ1 (Promega) for 30 min at 37 °C. Five hundred nanograms of RNA and DV2M3 and DV2M4 primers were used and the RT-PCR conditions were the same as those previously reported (Table S1 and [21]). To evaluate the RNA integrity, a reaction using primers for *Aedes aegypti* ribosomal S7 ribosomal protein (Table S1) was carried out under the same conditions. RNA from both C6/36 cells mock-infected and infected with DENV-2 at a multiplicity of infection (MOI) of 0.1 for 48 h were used as negative and positive controls, respectively. The products were analyzed in a 1% agarose gel in TBE buffer stained with Gel Red (Biotium).

### 2.5. Evaluation of Viral Interference by Cytopathic Effect

To evaluate homologous viral interference, C6/36 or C6-L cells were seeded in a 6-well plate (2 × 10^6^ cells per well) and incubated at 35 °C overnight to allow for adhesion. Then, the culture medium was removed and replaced with 1 mL of PBS supplemented with 0.5% fetal bovine serum with or without DENV-2 at an MOI of 0.5 and incubated for 1 h at 37 °C. Afterward, the cellular monolayers were washed twice with fresh culture medium to remove the excess virus, and 5 mL of new medium was added. The infection was allowed to proceed for 6 days at 35 °C, and the monolayers were observed with a Nikon Eclipse 80i microscope and photographed with a Nikon digital camera and Nikon ACT-2U software (https://act2u.software.informer.com/, accessed on 11 September 2005, version 1.6). Finally, the supernatant was removed, and the monolayers were fixed with 10% trichloroacetic acid (Sigma) for 5 min at room temperature and stained with 0.1% crystal violet (Sigma) in 20% ethanol (JT Baker) for 15 min at 37 °C.

### 2.6. Viral Interference Assay

To confirm homologous viral interference, C6/36 or C6-L cells were seeded in a 96-well plate (5 × 10^4^ cells per well) and infected (C6/36) or reinfected (C6-L) with DENV-2 at different MOIs. At several days post infection, the supernatant was recovered and used in a plaque assay in BHK-21 cells to determine the virus titer. Mock-infected C6/36 and C6-L cells were used as controls. The experiments were performed three times for each virus. To evaluate heterologous viral interference, a similar procedure was carried out using SINV.

### 2.7. Flow Cytometry Assays

To evaluate heterotypic and homologous viral interference, a flow cytometry assay was performed. A total of 6 × 10^5^ C6/36 or C6-L cells per well were seeded in 12-well culture plates and incubated at 35 °C for 24 h. Then, the cell monolayers were washed once with PBS supplemented with 0.5% bovine fetal serum and infected with either DENV or YFV at an MOI of 0.2 for 1 h at 37 °C with gentle shaking. Then, the cell monolayers were treated for 30 s with acid glycine (13.6 mM NaCl, 0.50 mM KCl, 0.05 mM MgCl_2_, 0.07 mM CaCl_2_, 10 mM glycine pH 2.8–3) and washed 5 times with PBS containing 0.5% bovine fetal serum. One fresh culture medium per well was added, and 48 (for DENV-2, -3 and -4) or 96 (for DENV-1) h post infection at 35 °C, the cells were processed for flow cytometry as previously described [26] using the following specific anti-virus antibodies: SC 65724 (Santa Cruz Biotechnology) diluted 1:100 for DENV-1, MAB 8702 (Millipore) diluted 1:100 for DENV-2, MAB 8703 (Millipore) diluted 1:200 for DENV-3, MAB 8704 (Millipore) diluted 1:600 for DENV-4, and ab36055 (Abcam) diluted 1:2500 for YFV. Then, a secondary antibody coupled to FITC (Invitrogen 62-6511) diluted 1:1000 was added. The experiments were performed twice in triplicate, and 10,000 events were counted in a FACSCalibur flow cytometer (Becton Dickinson) at Centro de Investigación y de Estudios Avanzados. The results were analyzed with Kaluza software (Beckman Coulter, Inc. https://www.beckman.mx/flow-cytometry/software/kaluza, accessed on 18 July 2023, version 2.2).

### 2.8. Silencing of Argonaute 3 Protein

To evaluate the participation of the genome-derived P-element-induced wimpy testis (PIWI) pathway in viral interference, an siRNA against *Aedes albopictus* Argonaute 3 (Ago3) mRNA was designed using i-Score Designer software (https://www.med.nagoya-u.ac.jp/neurogenetics/i_Score/i_score.html, accessed on 30 September 2018, version 1.1) and synthetized by Dharmacon^TM^ (Horizon-PerkinElmer). Additionally, an siRNA against green fluorescent protein (GFP) was used as a control. C6-L cells were seeded in a 6-well cell culture plate (1 × 10^5^ cells per well) and incubated at 35 °C overnight. Then, the culture medium was replaced, and the cells were transfected for 48 h with different amounts of siRNAs (0.8, 1, 1.5, and 2 µg) according to previous reports [27], using Lipofectamine^TM^ 3000 (Invitrogen) and following the manufacturer’s instructions. The silencing of Ago3 was evaluated via RT-qPCR. Briefly, total RNA was purified by RNAzol^®^ (MRC) according to the manufacturer’s instructions and treated with DNase (Thermo Scientific, Waltham, MA, USA). The cDNA was synthetized using the High Capacity cDNA Reverse Transcription kit (Thermo Fisher). For real-time PCR, TaqMan Universal PCR Master mix (Applied Biosystems, Waltham, MA, USA) was used, and the TaqMan probes are shown in Table S1. The ribosomal gene S7 was used as a loading control. The reactions were performed in an Mx3005P thermocycler (Agilent Technologies, Santa Clara, CA, USA), and the relative expression of Ago3 was evaluated using the 2Δ Ct formula. For the interference assays, the cells were infected with either DENV-2 or SINV at an MOI of 1 for 1 h and then transfected with siRNAs as described above. The viral titers and Ago3 silencing were evaluated 48 h after transfection.

### 2.9. Cell Viability Assay

To evaluate the toxicity of Lipofectamine and siRNAs, a similar experiment for Ago3 silencing was performed in a 96-well cell culture plate with 2.5 × 10^4^ cells per well. Cell viability was evaluated using the MTT Cell Proliferation I kit (Roche) according to the manufacturer’s instructions, and the absorbance was read at 594 nm in a Multiskan™ FC spectrometer (Thermo Scientific).

### 2.10. Statistical Analysis

The data were expressed as the mean ± SE and analyzed using Prisma Graph Pad 5 software (https://www.graphpad.com/features, accessed on 10 December 2015, version 6.07). Student’s multiple *t* tests or 2-way ANOVA with Holm–Sidak or Dunnett’s multiple comparison tests were used. A *p* < 0.05 was considered significant.

## 3. Results

### 3.1. Assessment of Viral Interference in C6/36 Cells Persistently Infected with DENV-2

One of the best models with which to study viral interference is persistently infected cell lines. In our laboratory, we have developed a C6/36 cell line from *Aedes albopictus* [22,23] persistently infected with DENV-2 (New Guinea C strain) that does not display cytopathic effects. After week 42 in culture, no virus particles were detected in the culture medium via plaque assay in BHK-21 cells [21], apparently because they release viral particles with restricted replication capacity [28]. RT-PCR was used confirm the presence of DENV infection in the C6/36 cell line persistently infected with DENV-2 (C6-L cell line), as described in the Section 2 (Figure S1).

**Figure 1:**
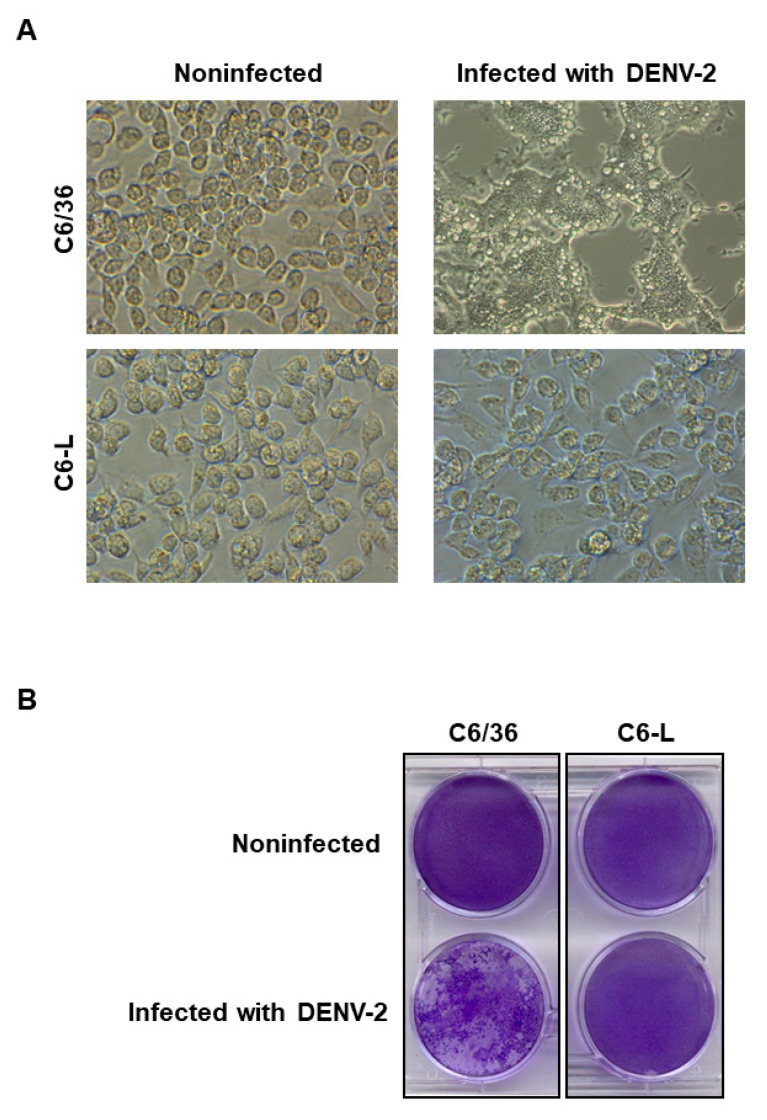
Analysis of viral interference by evaluating the cytopathic effect of cells infected with DENV-2. C6/36 or C6-L cell monolayers were infected (C6/36) or reinfected (C6-L) with DENV-2 at an MOI of 0.5 for 1 h. Then, the cells were washed to remove the excess virus, and six days after infection, the monolayers were observed with light microscopy at 40× (**A**). Finally, the supernatant was removed, and the monolayers were fixed with 10% TCA and stained with 0.1% crystal violet for 15 min at 37 °C (**B**).

Previous reports have shown that C6/36 cells either persistently [10] or acutely infected [5] with DENV display viral interference. To test whether the C6-L cell line shared the same characteristics, it was infected with DENV-2 at an MOI of 0.5, and the cytopathic effect was macro- and microscopically evaluated. C6/36 cells infected with DENV-2 clearly exerted a cytopathic effect characterized by syncytium formation, as previously reported [17,21,29] (Figure 1A), and the loss of monolayer integrity was evident when crystal violet staining was used (Figure 1B). Interestingly, when C6-L cells were reinfected with DENV-2 under the same conditions as C6/36 cells, neither a cytopathic effect (Figure 1A) nor monolayer destruction (Figure 1B) was observed. Instead, the cell monolayers resembled the appearance of the noninfected monolayers, suggesting the presence of viral interference.

**Figure 2:**
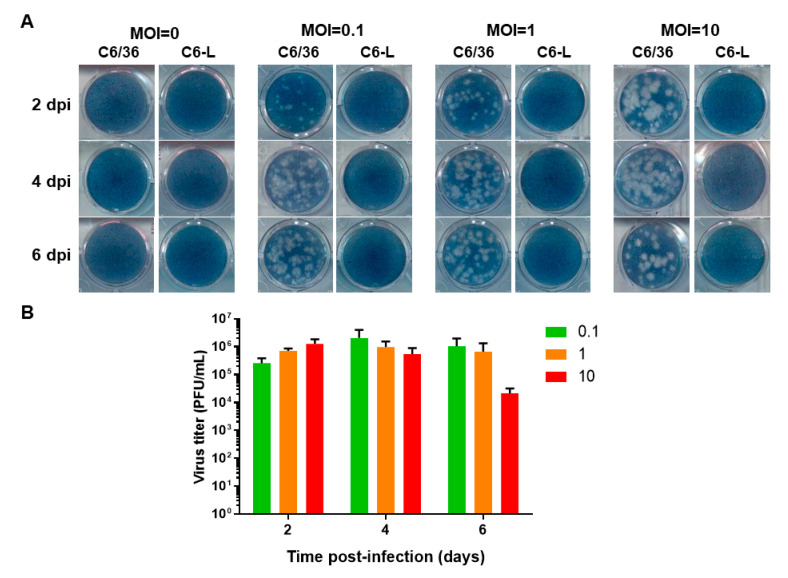
Assessment of viral interference at different times and MOIs. Monolayers of C6/36 or C6-L (persistently infected with DENV-2, passage 50) were infected (C6/36) or reinfected (C6-L) with DENV-2 at MOIs of 0.1, 1, and 10 for 1 h. Then, the cells were washed to remove the excess virus, and 2, 4, and 6 days after infection (dpi), the supernatant was recovered and used to titer the virus via plaque assay in BHK-21 cells. MOI = 0 represents mock-infected cells. (**A**) Photographs of BHK-21 monolayers stained with naphthol blue black showing the lytic plaques. (**B**) Viral titer in C6/36 cells infected with DENV-2. The experiment was performed in triplicate, and the mean ± SE is shown. The results were analyzed via two-way ANOVA and Tukey’s multiple comparisons test. No significant differences were found.

To determine whether the viral interference displayed by C6-L was dose- and time-dependent, BHK-21 cells were reinfected with DENV-2 at different MOIs (0.1, 1 and 10) for different periods of time (2, 4, and 6 days), and the virus titer in the culture medium was evaluated via plaque assay. C6/36 cells were used as positive controls (Figure 2).

No lytic plaques were observed at an MOI of 0, even if undiluted culture medium was used to perform the plaque assay in C6-L cells (Figure 2A), because they do not release infectious viral particles after passage 42, even when the viral genome is detected via RT-PCR, which has been previously reported [21].

Interestingly, when C6-L cells were reinfected with DENV-2 at different MOIs for different periods of time, no lytic plaques were observed in any case, even if undiluted culture medium was used in the plaque assay. In contrast, DENV-2 established a productive infection in C6/36 cells, as evidenced by lytic plaque formation (Figure 2A), which could be used to determine the viral titer over time (Figure 2B).

### 3.2. Assessment of Homologous Viral Interference in C6/36 Cells Persistently Infected with DENV-2

**Figure 3:**
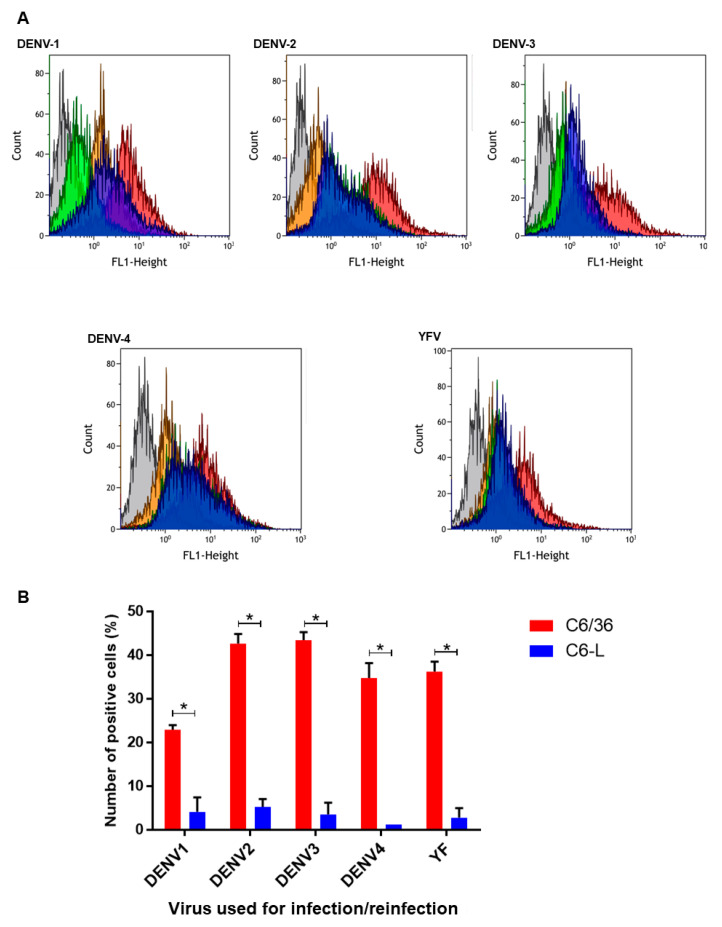
Determination of viral interference via flow cytometry. C6/36 (C6) or C6-L (CL) cells were independently infected (C6) or reinfected (CL) with the four DENV serotypes or YFV at an MOI of 0.2 for 1 h. After 48 (DENV 2–4 and YFV) or 96 (DENV 1) h, the cells were processed for flow cytometry using specific DENV (for each serotype) or YFV antibodies and a secondary antibody coupled to FITC. (**A**) Histograms of a representative experiment. The virus used for infection is indicated in each histogram. The background (gray) corresponds to cells not incubated with antibodies. C6/36 cells noninfected (orange); C6-L cells nonreinfected (green); C6/36 cells infected (red); and C6-L cells reinfected (blue) with DENV or YFV. (**B**) Graphic representation of one experiment performed in triplicate. The background obtained from noninfected C6/36 or C6-L nonreinfected cells incubated with both antibodies was subtracted. The mean ± SE is shown in each bar. Multiple *t* tests with Holm–Sidak’s multiple comparisons tests were performed with GraphPad Prism. * *p* ≤ 0.0009.

The experiments shown in Figure 2 suggested that the C6-L cell line had developed viral interference against DENV-2. To evaluate whether this phenomenon occurred in cells infected with the other DENV serotypes, monolayers of C6-L cells were separately infected with DENV-1 to DENV-4 for 1 h at an MOI of 0.2. Then, the cells were treated with acid glycine to inactivate the extracellular virus, and 48 h post infection, they were harvested and processed for flow cytometry using specific monoclonal antibodies against each serotype (Figure 3A and Figure S2). In the case of DENV-1, it was necessary to perform the analysis at 96 h post-infection to obtain a similar percentage of control-positive cells as that with the other serotypes. Again, C6/36 cells were used as positive controls, and as shown in Figure 3B (red bars), they were effectively infected in all cases. In contrast, C6-L subjected to the same virus dose and infection procedure barely reached 8% of positively infected cells in the case of DENV-1 and approximately 2–4% for the other serotypes. In the case of DENV-2, the antibody was able to recognize the virus present in noninfected cells because of the persistent infection, but reinfection with the same serotype only slightly increased the number of positively infected cells. In all cases, the difference between C6/36 and C6-L cells infected with the same serotype was statistically significant, with *p* ≤ 0.0009. Although the degree of refractory infection displayed by C6-L cells was apparently greater for DENV-4 and smaller for DENV-1 (Figure 3B), the differences were not significant. Altogether, these results suggest that C6-L cells display heterotypic viral interference against all four DENV serotypes. To determine whether this phenomenon extended to other flaviviruses, we performed the same experiment described above but with YFV instead. Interestingly, the same effect was observed in C6-L cells infected with YFV at an MOI of 0.2, where only 2.5% of the cells were positive for the infection, whereas the C6/36 cells infected under the same conditions reached almost 50% positivity (Figure 3). Again, this difference was statistically significant (*p* ≤ 0.0009).

### 3.3. Assessment of Heterologous Viral Interference in C6/36 Cells Persistently Infected with DENV-2

**Figure 4:**
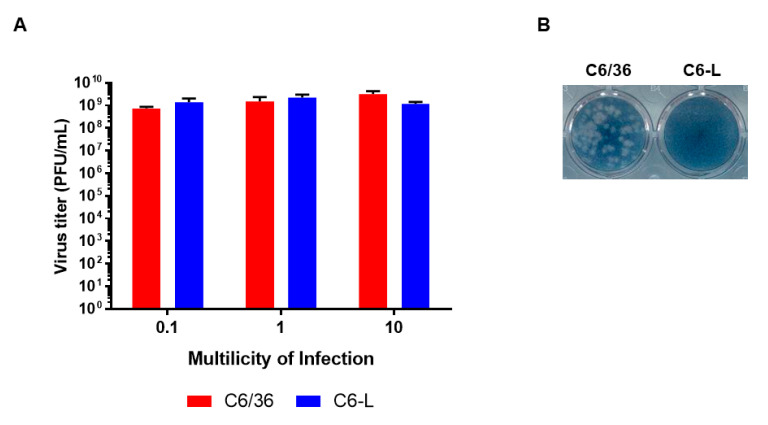
Assessment of heterologous viral interference in C6/36 cells persistently infected with DENV-2. Monolayers of C6/36 or C6-L were infected with SINV at different multiplicities of infection for 1 h. Then, the cells were washed to remove the excess virus, and two days after infection, the virus titer was determined in the supernatant via plaque assay in BHK-21 cells. The experiments were carried out twice in triplicate, and a representative experiment is shown (**A**). The mean ± SE is shown in each bar. The results of the infection with DENV-2 at an MOI of 1 for two days were included as a control (**B**). The results were analyzed via one-way ANOVA with Holm–Sidak’s multiple comparisons test. Nonsignificant differences were found.

To evaluate whether the interference could be extended to other arboviruses, a similar experiment using different MOIs of SINV was performed, and the viral titers were evaluated via plaque assay. As shown in Figure 4A, the SINV titers were similar in C6/36 and C6-L; however, C6-L still retained viral interference against DENV-2 (Figure 4B). Altogether, these results suggest that C6-L cells display homologous but not heterologous viral interference.

### 3.4. Participation of the Genome-Derived P-Element-Induced Wimpy Testis (PIWI) Pathway in Viral Interference

The importance of the small interfering RNA (siRNA) response in antiviral defense in insects has been widely discussed in the literature, and its importance is probably associated with viral interference as well. Previous analysis of the small RNA population in C6/36 cells infected with several arboviruses suggests that the PIWI pathway might participate in viral interference [30]. Since Ago3 protein has a relevant function in this pathway [31], we selected it for silencing.

**Figure 5:**
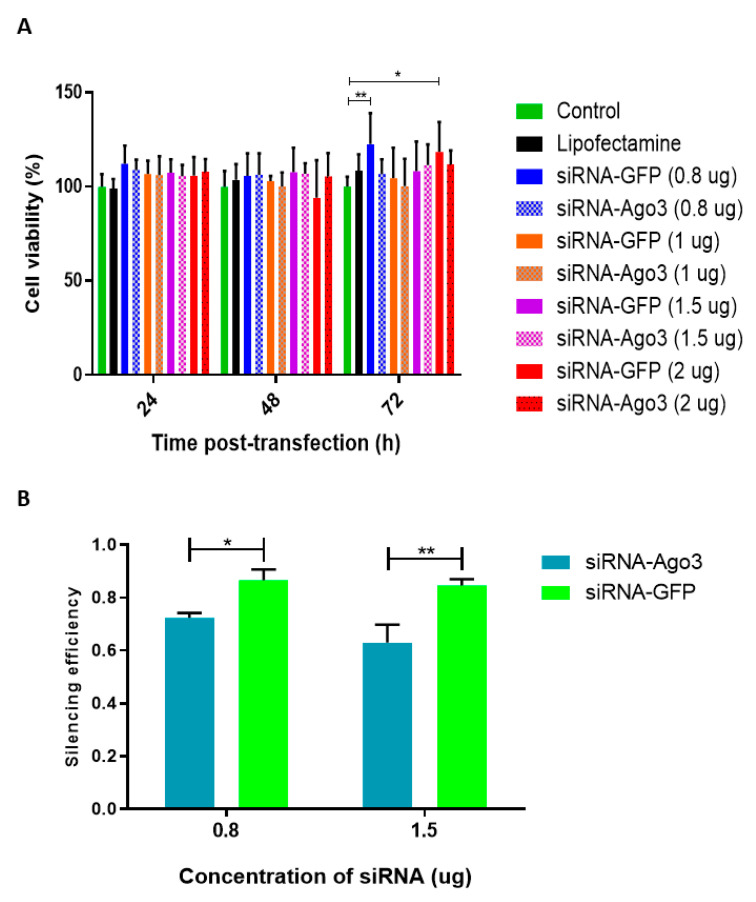
Assessment of cell toxicity and silencing efficiency of siRNA against Ago3. (**A**) C6-L cells were transfected with different amounts (0.8–2 µg) of either siRNA against Ago3 (siRNA-Ago3) or against green fluorescent protein (siRNA-GFP) as a control. At 24, 48, and 72 h after transfection, cell viability was evaluated via MTT assay. The assay was performed twice in triplicate, and all results are shown. The results were analyzed via two-way ANOVA and Dunnett’s multiple comparison test. * *p* = 0.003, ** *p* = 0.006. (**B**) Ago3 was silenced using either siRNA-Ago3 or siRNA-GFP in C6-L cells. The effects of different concentrations of siRNAs (0.8 and 1.5 µg) were evaluated via RT-qPCR. The results were analyzed via Student’s *t* test and Holm–Sidak’s multiple comparisons test. * *p* = 0.04, ** *p* = 0.006.

C6-L cells were transfected with different concentrations (0.8, 1, 1.5, and 2 µg) of a specific siRNA against Ago3 mRNA. An siRNA against GFP was included as a control. The toxicity of Lipofectamine and siRNAs in C6-L cells was evaluated via MTT assay. No evidence of cytotoxicity was found (Figure 5A).

The concentrations of siRNA that achieved the best reduction in the levels of Ago3 mRNA were 0.8 µg (reduction of 28%) and 1.5 µg (reduction of 37%) (Figure 5B).

**Figure 6:**
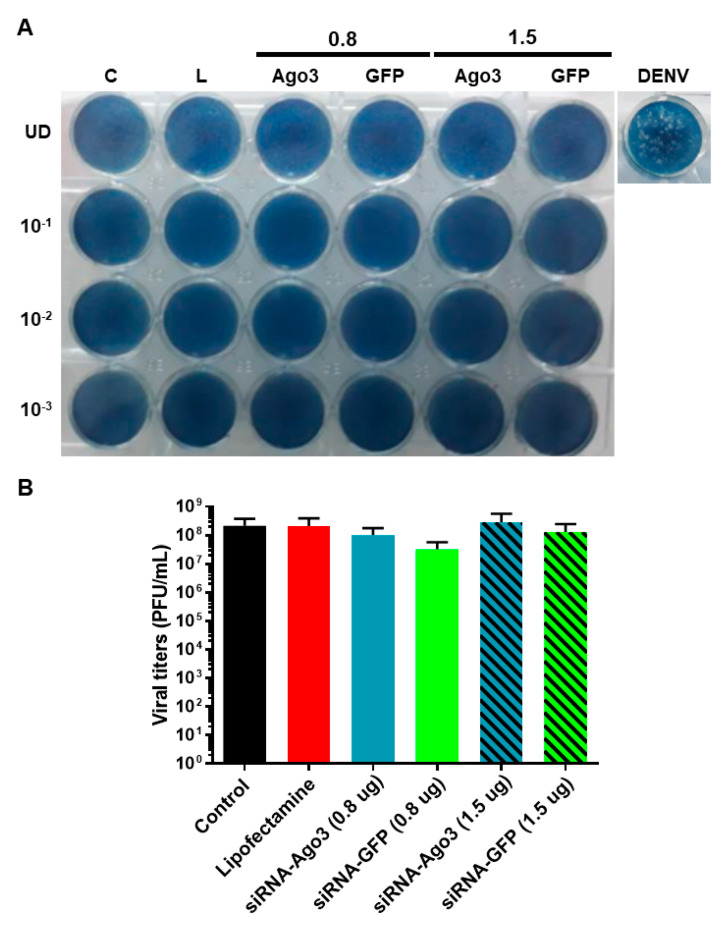
Participation of Ago3 in viral interference. C6-L cells were transfected with 0.8 or 1.5 µg of siRNA against Ago3 or GFP as a control. Then, they were reinfected with DENV-2 or SINV at an MOI of 1 for 48 h. Viral titers were determined via plaque assay in BHK-21 cells. Plaque assay using DENV-2 stock is shown as a control (DENV). A representative plaque assay for DENV is shown in (**A**). C (control), nontransfected cells; L, cells treated with Lipofectamine without siRNA; Ago3, cells transfected with siRNA against Ago3; GFP, cells transfected with siRNA against GFP. Titration of C6-L cells infected with SINV was performed twice in triplicate, and a representative experiment is shown in (**B**). The results were analyzed via one-way ANOVA and Tukey’s multiple comparisons test. Nonsignificant differences were found.

However, the silencing of Ago3 did not have any effect on homologous interference against DENV-2 since no viral titers were detected after reinfection for 48 h at an MOI of 1 (Figure 6A). Moreover, the infection of C6-L cells with SINV at an MOI of 1 for 48 h remained intact after the silencing of Ago3 (Figure 6B). Altogether, these results suggest that the viral interference displayed by C6-L cells is apparently PIWI-independent.

## 4. Discussion

In our laboratory, we developed a C6/36 cell line persistently infected with DENV-2 (New Guinea strain) based on the protocol reported previously by Igarashi, which displays characteristics such as the absence of cytopathic effects and low viral titers [10,18,20,21].

C6-L cells showed a substantial reduction in virus yields when they were independently reinfected with all DENV serotypes. This effect was stronger when passages over 50 were used, indicating the presence of heterotypic viral interference. Although we did not establish C6/36 cell lines persistently infected with the other DENV serotypes to test cross interference, previous reports indicate that heterotypic viral interference is a common phenomenon in persistently infected C6/36 cells [10] and acutely infected C6/36 cells [5]. This phenomenon was confirmed in cells infected with all four serotypes via flow cytometry. Compared with the nonpersistently infected cells, the C6-L cells that were reinfected with the respective DENV serotype showed only a slight increase in the number of cells positive for specific viral antigens. Similar results have been obtained in C6/36 cells persistently infected with *Aedes albopictus* densovirus (A*al*DNV), reinfected with the same virus, and evaluated via flow cytometry [3].

Homologous viral interference is a common feature during flavivirus infections. For example, Vero cells persistently infected with MVE (strain OR2) displayed viral interference when they were reinfected with the same virus (strain OR155) or other flaviviruses, such as Kunjin (KUN), YFV, or WNV [32]. Similar results have been obtained with Vero [33,34], MA-111 [33], and KN73 [35] cells persistently infected with JEV. This phenomenon has been reported for other arboviruses, such as Bunyamwera [14], Banzi [12,13], SFV [2], CHIKV [10], and SINV [4,7,8,9]. Additionally, homologous interference has been observed in vivo. For example, *Aedes triseriatus* mosquitoes sequentially infected via the intrathoracic route with different members of the *Buyaviridae* family displayed resistance to the second inoculated virus, but this phenomenon was not observed when the second infection was with heterologous viruses such as vesicular stomatitis virus (VSV) or WNV [36]. C6-L cells displayed homologous viral interference, at least against YFV, which was similar to Vero cells persistently infected with MVE [32].

Interestingly, the C6-L cells were able to be reinfected with an alphavirus such as SINV, indicating the absence of heterologous interference. This finding is in accordance with previous reports revealing that C6/36 cells, persistently infected with all four DENV serotypes independently, are still susceptible to CHIKV replication [10], and that C6/36 cells persistently infected with DENV-2 are susceptible to reinfection with A*al*DNV [20]. The absence of heterologous viral interference has also been reported in C6/36 cells persistently infected with other viruses, such as Bunyamwera [14] and SINV [4]. These cells are still susceptible to infection with Dugbe virus (Nairovirus), VSV [14], or WNV [4]. Simultaneous infection or coinfection of C6/36 cells with Densonucleosis virus (DNV) and CHIKV does not result in interference with CHIKV replication [20]. This absence of interference has also been reported in other cells persistently infected with different viruses, such Banzi [12,13], SLE [17], JEV [33,34], and SINV [4,7,8], and observed in *Aedes* mosquitoes in vivo [36].

The mechanisms involved in viral interference during flavivirus and alphavirus infections have not been completely elucidated. Some lines of research suggest that factors of viral origin may play a role [2,5,8,11,33,34], and the presence of homologous and absence of heterologous viral interference may be due to different requirements of cellular and viral factors to complete their replicative cycles [1,20,34]. However, cellular factors are not totally excluded from the viral interference mechanism [8,37,38,39,40]. Recently, it has been proposed that endogenous LTR retrotransposons together with the RNAi machinery present in *Drosophila melanogaster* S2, *Aedes albopictus* C6/36 and U4.4, and *Aedes aegypti* Aag2 cell lines are responsible for the establishment and maintenance of persistent viral infections. The mechanism includes the retrotranscription of the viral RNA genome into viral DNA (vDNA), which is, in turn, transcribed to synthesize double-stranded RNAs (dsRNAs) that can be used by Dicer-2/Ago2 to generate small viral RNAs that degrade the viral genome and control viral replication [41,42]. Moreover, *Aedes aegypti* mosquitoes with a mutation in exon 5 of Dicer-2 display higher viral loads and mortality after SINV, YFV, DENV-2, and DENV-4 infections [43]. This mechanism apparently induces host tolerance to arbovirus infection, and it might be responsible for the homologous and heterotypic viral interference and the absence of heterologous viral interference observed in C6-L cells. However, analysis of the small RNA population in C6/36 cells infected with WNV, SINV or LACV revealed that this cell line generates viRNAs in a Dicer-2-independent manner, suggesting that it has a defect in the canonical RNAi machinery [30]. This finding was later corroborated in C6/36 cells infected with DENV, where the viRNA population was predominantly 27 nt in length and derived from the positive-sense viral genome. Further studies revealed that C6/36 cells can assemble the RISC complex and have reduced Dicer-2 activity, apparently because a mutation generates a stop codon in the mRNA transcript [27]. In contrast, uninfected C6/36 cells express an important population of piRNAs [44]. Similar results have been observed in *Aedes albopictus*-derived C7-10 cells infected with CHIKV, which display a deletion of 33 amino acids in Dicer-2 [45].

piRNAs are one of the endogenous RNA interference (RNAi) pathways in insects [46]. These small RNAs are single-stranded and 24–30 nt in length, with a sequence bias for uridine at the 5′ end (U1). In *Drosophila melanogaster*, they participate in the control of transposon expression in the germ line at the transcriptional and posttranslational levels. In mosquitoes, they are expressed in somatic tissues and have an expanded repertoire that includes viral sequences. They are first generated as intermediate piRNAs by an endonuclease (Zucchini) from an antisense RNA precursor transcribed from genomic transposon-rich clusters, piRNA clusters, or mRNAs. This intermediate binds to the PIWI family proteins and is trimmed and 2′ O-methylated. PIWI-loaded piRNAs participate in the transcriptional silencing of transposons and are also cleaved to generate secondary piRNAs that bind to the Ago3 protein. These secondary piRNAs are positive sense and have an adenine in position 10 (A10). They can hybridize with transcripts generated from piRNA clusters, which are cleaved to generate a new piRNA in a process referred to as the “ping-pong” amplification cycle [47,48,49]. In *Aedes aegypti*, Ago3 and Piwi1-7 proteins participate in the piRNA pathway, where Piwi1–Piwi3 are germline specific [50,51]. In *Aedes albopictus*, nine PIWI proteins have been reported: Piwi1-9 and Ago3 [52,53].

The generation of vpiRNAs has been reported in mosquitoes or mosquito-derived cell lines infected with various flaviviruses and alphaviruses, such as U4.4 (from *Aedes albopictus*) cells infected with CHIKV [42], SINV [31], DENV-2 [54], and WNV [44]; C6/36 cells infected with DENV-2 [54], SINV [31,55], or CHIKV [45]; and Aag2 (from *Aedes aegypti*) cells infected with DENV-2 [54], WNV [44], ZIKV [56], SINV [31], and SFV [57]. In *Aedes albopictus* female mosquitoes, an upregulation in piRNAs after infection with DENV-2 [52] or CHIKV [45,58] has been reported. *Aedes aegypti* mosquitoes infected with DENV-2 [59], SINV, YFV [43], RRV [60], and CHIKV [45] also display an increase in piRNA response. Moreover, Piwi4 silencing in either Hsu or CT (from *Culex* mosquitoes) cells resulted in increased levels of Merida virus (MERDV) or LACV viral RNA [61]. Similar results have been observed in Aag2 cells infected with ZIKV [56] or SFV [57] after silencing Piwi4, suggesting the participation of the piRNA pathway in the host antiviral response and probably in viral interference.

In this work, after silencing Ago3, a key protein in piRNA biogenesis, in C6-L cells, neither homologous nor heterologous viral interference was modified. One explanation is that the reduction in Ago3 mRNA levels was not enough to have a substantial impact on protein levels, and since there are no commercial antibodies against mosquito Ago3 protein available, this issue shall be analyzed in more detail. However, recent evidence suggests that the piRNA pathway might not participate in the antiviral response or that it is secondary. For example, silencing Piwi4 in Aag2 cells infected with SFV increased viral titers and replication; however, this effect is apparently independent of Dicer-2 and piRNA production [57]. *Aedes aegypti* mosquitoes with a loss of Dicer-2 function are still able to synthetize vpiRNAs, but they do not survive after SINV, YFV, DENV-2, or DENV-4 inoculations and display an increase in viral load [43]. Moreover, silencing of Ago3 expression in CT cells did not have any effect on the levels of viral RNA of Charoen-like (PCLV), Calbertado (CLBOV), LACV, or Usutu (USUV) viruses [61], and silencing of Ago3 or Piwi5 in Aag2 cells that do not express Dicer-2, and that consequently produce high levels of piRNAs, do not display an increase in viral titers of SFV [57].

A population of sRNAs that is 19 nt in length that mapped to the S segment of the sense strand has been detected in Hsu cells infected with LACV [61], and *Aedes albopictus* female mosquitoes infected with DENV-2 display an upregulation in piRNAs but without the ping-pong signature, suggesting an unknown alternative biogenesis pathway in mosquitoes infected with flaviviruses [52]. Additionally, microRNAs (miRNAs) that target several arbovirus genomes [62] and tRNA fragments (tRF) that are modulated in mosquitoes during DENV infection have been identified [63]. However, whether these small RNAs have a role in the antiviral response and interference will require further investigation.

In addition to RNA machinery, other factors could be involved in viral interference. For example, it has been reported that several ABC transporters, a family of proteins with multiple functions, are differentially expressed in *Aedes aegypti* mosquitoes and Aag2 cells infected with several flaviviruses, such as DENV, YFV, and CHIKV. Interestingly, the promoters of these genes have response elements to STAT and Rel1 transcription factors, both of which are involved in innate immunity in mosquitoes, suggesting their participation in viral infection [64]. Moreover, peroxidases are involved in the immune response, and it has been reported that the *Anopheles* HPX15 peroxidase is overexpressed and secreted in the midgut during blood feeding, inducing the formation of a physical barrier between food particles and the epithelium and the suppression of midgut immunity. They also have response elements to STAT and Rel1 transcription factors in their promoters [65]. Since arboviruses first contact their vector in the midgut, these peroxidases might participate in the antiviral response. However, the role of these factors in viral interference will also require further investigation.

## 5. Conclusions

In conclusion, C6/36 cells persistently infected with DENV-2 display viral interference against the four DENV serotypes and YFV, but they can support SINV replication, suggesting the presence of heterotopic and homologous viral interference but the absence of heterologous viral interference. Although several lines of research suggest that the Dicer-2-independent small RNA-interfering pathway might be responsible for viral interference, the silencing of Ago3, a key protein in the PIWI pathway, did not have any effect, suggesting that other factors might be responsible.

## Data Availability

Data generated or analyzed during this study are included in this article and its supplementary material files. Further inquiries can be directed to the corresponding author.

## Supplementary Materials

The following supporting information can be downloaded at https://www.mdpi.com/article/10.3390/pathogens12091135/s1: Figure S1: RT-PCR to detect the DENV genome; Figure S2: Complementary flow cytometry assays; Table S1: Primers used for RT-PCR and qPCR.
