# Supplementary material for: Characterization of Viral Interference in Aedes albopictus C6/36 Cells Persistently Infected with Dengue Virus 2
Source: Pathogens. 2023 Sep 6;12(9):1135. doi: 10.3390/pathogens12091135 (PMC10536104; doi:10.3390/pathogens12091135)
Supplement: Supplementary file 1 [file pathogens-12-01135-s001.zip › Supplemenary figures.pdf]

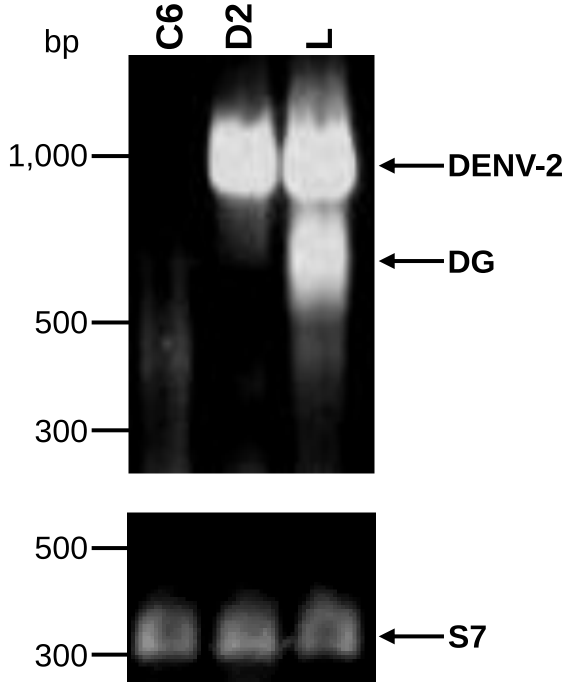

Supplementary Figure 1. RT-PCR to detect DENV genome**.** Five hundred nanograms of total RNA from mock-infected (C6), acutely infected with DENV-2 (D2) or C6-L (L) cells were used in a RT-PCR using the conditions reported previously (see Material and Methods). Primers for S7 ribosomal protein (S7) were used as control. The amplicons (indicated by arrows) were analysed in a 1% agarose gel in TBE buffer stained with Gel Red (Biotium). The molecular markers (100 bp ladder New England Biolabs) are indicated in the left side of the gel. DENV-2, wild type viral genome; DG, defective viral genome; S7, S7 ribosomal protein.

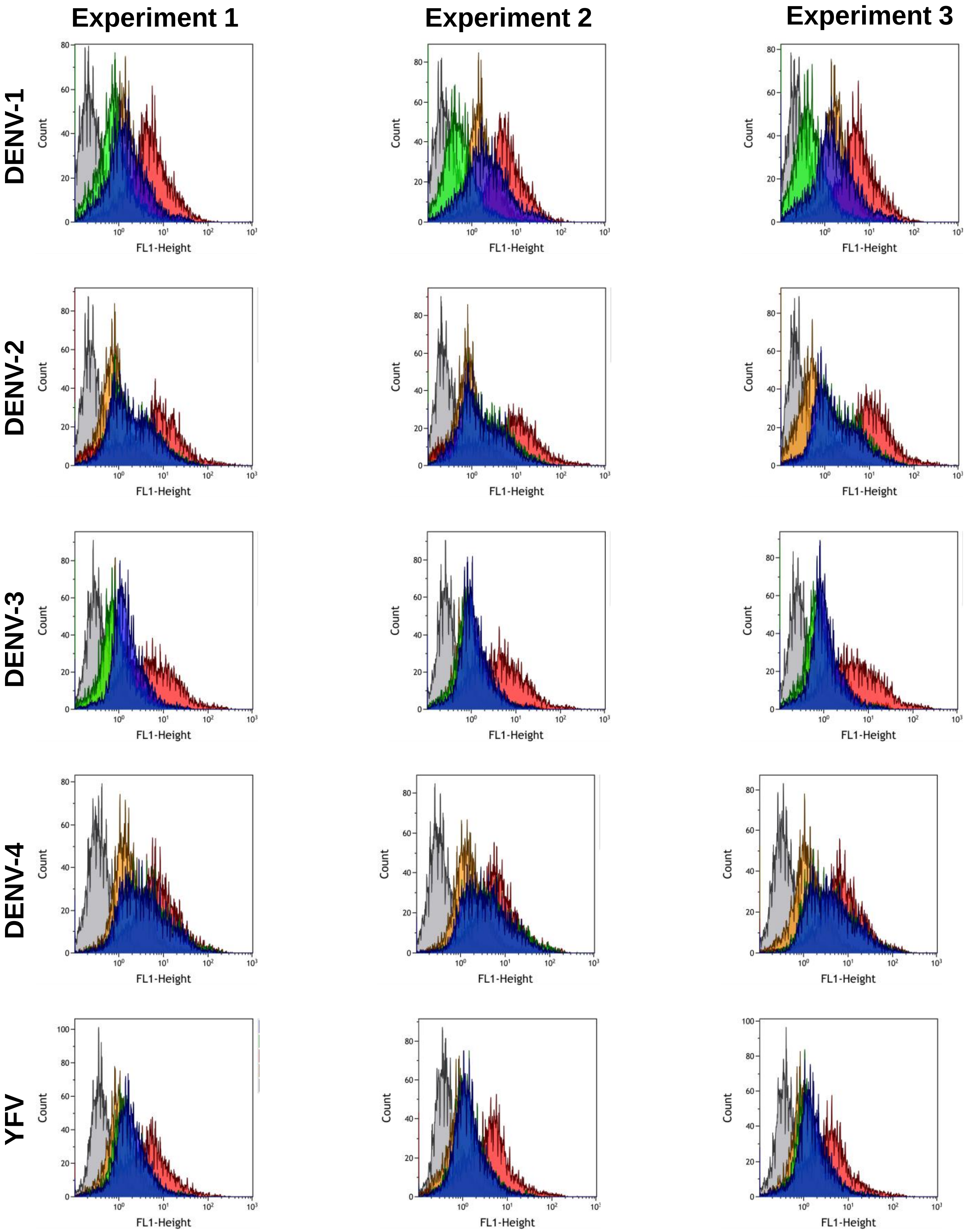

Supplementary Figure 2. Complementary flow cytometry assays. C6/36 (C6) or C6-L (C6L) cells were infected (C6) or re-infected (C6L) independently with the four DENV serotypes or YFV at a MOI of 0.2 for 1 h. Forty-eight (DENV 2-4 and YFV) or 96 (DENV 1) h, the cells were processed for flow cytometry using specific DENV (for each serotype) or YFV antibodies and a secondary antibody coupled to FITC. Histograms of a representative experiment performed in triplicate. Gray, cells non-incubated with antibodies; orange, non-infected C6/36 cells; red, C6/36 infected with DENV or YFV; green, C6-L cells non-reinfected; and blue, C6-L cells re-infected with DENV or YFV.
