# Supplementary material for: Characterization of Viral Interference in Aedes albopictus C6/36 Cells Persistently Infected with Dengue Virus 2
Source: Pathogens. 2023 Sep 6;12(9):1135. doi: 10.3390/pathogens12091135 (PMC10536104; doi:10.3390/pathogens12091135)
Supplement: Supplementary file 1 [file pathogens-12-01135-s001.zip › Table S1.pdf]

Supplementary Table 1:

Primers used for RT-PCR

Gen	Forward primer 5′-3′	Reverse primer 5′-3′
DENV	acaaaccaacattggattttgaact	agctgtctccgaatggaggttctgc
S7	gggacaaatcggccaggctatc	tcgtggacgcttctgcttgttg

DENV, Dengue virus; S7, ribosomal S7 gen.

Primers used for qPCR.

Gen	Forward primer 5′-3′	Reverse primer 5′-3′	TaqMan probe 5′-3′
Ago3	ccgaagcagtgtcacaggtc	gtcgcatgccagtcggatatag	cgggaaccagcggaactcccgttc
S7	atctggtcttcccggctgaag	ctgacgtgaaggtgtcgacc	gacggatcgcagctgatcaaggtgc

The TaqMan probe was labeled with FAM and BHQ1. Ago3, argonaute 3; S7, ribosomal S7 gen.
